# Post-stroke insomnia: multidimensional mechanisms, clinical heterogeneity, and toward mechanism-informed, objectively quantified management

**DOI:** 10.3389/fneur.2026.1820460

**Published:** 2026-05-04

**Authors:** Jinlian Liao, Jijie Peng, Yi Wang, Yan Wei, Jiamin Wu

**Affiliations:** 1 Zhuhai Hospital of Integrated Traditional Chinese and Western Medicine, Zhuhai, China; 2 Faculty of Chinese Medicine, Macau University of Science and Technology, Macau, China; 3 Zhongshan Hospital of Traditional Chinese Medicine, Zhongshan, China

**Keywords:** heterogeneity, hyperarousal, neuroinflammation, post-stroke insomnia, stratified management

## Abstract

Stroke survivors frequently experience sleep disturbance, among which post-stroke insomnia (PSI) is common yet often underrecognized across the post-stroke course. Although its clinical expression may vary over time, PSI is associated with reduced rehabilitation engagement, impaired quality of life, and potentially adverse long-term outcomes. Accumulating evidence suggests that PSI is not a unitary entity; rather, it reflects interacting neurobiological and psychosocial processes, including injury to sleep–wake regulatory networks, neurotransmitter and circadian disruption, neuroinflammation, hypothalamic–pituitary–adrenal (HPA) axis and autonomic hyperarousal, hemodynamic and neurovascular dysfunction, and comorbid conditions such as pain, nocturia, and sleep-disordered breathing. Despite growing interest, PSI management in clinical practice largely follows general insomnia strategies, and interpretation of treatment effects is constrained by heterogeneous intervention protocols, limited objective sleep assessment, and short follow-up. Methods: This narrative review was informed by searches of PubMed, Web of Science, Google Scholar, and Chinese databases (CNKI and Wanfang) from inception to February 2026, complemented by reference screening. This review synthesizes key mechanistic domains and sources of heterogeneity in post-stroke insomnia (PSI) and discusses a pragmatic, mechanism-informed approach emphasizing objective sleep phenotyping and coordinated management of dominant drivers. We highlight controversies, current research gaps, and near-term opportunities to advance PSI care through standardized definitions, combined subjective–objective outcomes, and stratified interventions aligned with patient-level mechanisms.

## 1 Introduction

Stroke remains a leading cause of death and long-term disability, and the expanding population of stroke survivors has shifted clinical priorities toward sustained functional recovery and quality-of-life management (1, 2). Sleep disorders are prevalent after stroke and may be underrecognized amid motor, speech, cognitive, and emotional sequelae. Among these conditions, PSI—new-onset or clearly worsened persistent insomnia symptoms after a cerebrovascular event—often presents with difficulty initiating sleep, difficulty maintaining sleep, or early morning awakening, accompanied by daytime impairment (3, 4).

Stroke is inherently heterogeneous in etiology, lesion patterns, vascular burden, and clinical course. In this review, the term “stroke” is used broadly to encompass both ischemic and hemorrhagic stroke. Differentiation of stroke subtypes (e.g., cardioembolic, lacunar/small-vessel disease–related infarction, atherothrombotic infarction, infarcts of other determined causes, and intracerebral hemorrhage) is clinically meaningful because it relates to risk-factor distributions, stroke severity, prognosis, and downstream complications. However, sleep–wake manifestations may vary across stroke subtypes and stages; in particular, acute intracerebral hemorrhage may more often present with impaired consciousness or hypersomnolence than with insomnia, whereas insomnia-related complaints may become more relevant beyond the acute phase in some patients. This heterogeneity may also shape PSI phenotypes and treatment response through differences in lesion topography, comorbidity profiles (e.g., sleep-disordered breathing, autonomic dysregulation), and the broader vascular and inflammatory milieu (5, 6).

Importantly, PSI should not be viewed as a single-cause complaint. Stroke survivors frequently face concurrent mood symptoms, pain, urinary symptoms, environmental disruption during hospitalization or rehabilitation, and sleep-disordered breathing, all of which may interact with stroke-related neurobiological changes (7). This multidimensional etiology likely contributes to marked interindividual differences in symptom profiles and treatment response. Meanwhile, clinical management frequently extrapolates from general insomnia practice, which may be limited in stroke populations by comorbidity burden, functional constraints, and insufficient objective quantification of sleep changes (8).

Given the heterogeneity in PSI definitions, outcome measures, and study designs, this article is intended as a narrative review rather than a formal systematic review or meta-analysis. To support the synthesis, we searched PubMed, Web of Science, Google Scholar, and the Chinese databases CNKI and Wanfang (from inception to February 2026). Searches were built around the core concepts of “stroke/post-stroke” and “insomnia/sleep disturbance,” combined with terms capturing relevant mechanisms and phenotypes (e.g., sleep–wake regulation, circadian function/melatonin, inflammation, HPA-axis and autonomic arousal, sleep-disordered breathing, and actigraphy/wearable monitoring). Reference lists of key articles were also screened to identify additional pertinent studies. We prioritized population-based and clinical studies, as well as guidelines/consensus documents relevant to PSI, and organized the evidence by mechanistic domains to inform heterogeneity and management considerations.

This narrative review focuses on (i) core mechanistic domains that plausibly contribute to PSI, (ii) clinical heterogeneity and its implications for management, and (iii) a practical direction toward mechanism-informed, objectively quantified care.

## 2 Mechanisms and heterogeneity of post-stroke insomnia

PSI likely arises from interactions among biological and behavioral processes rather than from a single lesion location or pathway. Below, we summarize major mechanistic domains and how they may map onto heterogeneity in clinical presentation. Details are summarized in Table 1.

**Table 1:** Mechanistic domains, heterogeneity clues, and mechanism-informed management implications of post-stroke insomnia (PSI).

Mechanistic domain	Key pathophysiological features	Clinical manifestations/heterogeneity clues	Mechanism-informed management implications
Sleep–wake network injury	Lesions in thalamus/brainstem/basal ganglia; network disconnection/remodeling	Difficulty initiating sleep, sleep fragmentation, frequent awakenings; phenotype may vary by lesion site/stage	Integrate lesion location + stage + function into assessment; avoid symptom-only treatment; consider objective continuity metrics
Neurotransmitter and circadian dysregulation	5-HT/GABA/Glu imbalance; melatonin reduction; circadian misalignment	Prolonged sleep onset; irregular timing; mixed onset + maintenance complaints	Prioritize sleep timing/zeitgebers; consider melatonin/rhythm interventions where appropriate; use objective metrics (SOL/SE) to identify dominant pattern
Neuroinflammation/immune dysregulation	Elevated IL-6/TNF-α/CRP; inflammatory effects on sleep circuits	Increased awakenings; non-restorative sleep; persistence in some patients	Screen inflammatory/comorbidity burden; link mechanistic endpoints to sleep outcomes in studies; avoid overattributing causality without longitudinal evidence
Activation of the Stress System	Cortisol dysregulation; nocturnal sympathetic activation; sustained hyperarousal	Hyperarousal; light sleep predominance; frequent awakenings; daytime fatigue	Shift goals from “sleep promotion” to arousal reduction + continuity stabilization; prioritize behavioral treatment where feasible; combine behavioral components with careful medication selection; consider cautious, individualized, time-limited pharmacotherapy when appropriate; monitor daytime safety.
Neurovascular/hemodynamic vulnerability	Impaired perfusion regulation; endothelial dysfunction; neurovascular unit injury	Reduced sleep stability; often coexists with other domains	Emphasize multimodal interpretation (vascular + arousal + comorbidities); pair objective monitoring with mechanistic measures in research; avoid oversimplified causal claims
Psychosocial and comorbidity drivers	Emotional distress/rehab stress; pain/nocturia/SDB; environmental disruption	Onset/maintenance difficulty; repeated awakenings; SDB may coexist; chronicity tendency	Systematic screening + coordinated management of dominant drivers (pain, bladder symptoms, SDB, mood); stratify into emotion-dominant vs. comorbidity-dominant profiles for targeted care

5-HT, 5-hydroxytryptamine (serotonin); GABA, gamma-aminobutyric acid; Glu, glutamate; IL-6, interleukin-6; TNF-α, tumor necrosis factor-alpha; CRP, C-reactive protein; HPA, hypothalamic–pituitary–adrenal; SOL, sleep onset latency; SE, sleep efficiency; SDB, sleep-disordered breathing.

### 2.1 Lesion and network disruption in sleep–wake regulation

Sleep–wake regulation depends on distributed circuitry involving the hypothalamus, thalamus, basal forebrain, and brainstem ascending arousal system. Stroke affecting these nodes or their connections may impair sleep initiation, destabilize sleep–wake transitions, and increase nocturnal awakenings. Damage to sleep-promoting elements of this network, including the ventrolateral preoptic area (VLPO), may contribute to difficulty initiating sleep, poor sleep continuity, frequent nocturnal awakenings, and sleep fragmentation (9). In stroke populations, however, these features are more likely to reflect disruption of the broader sleep-promoting network rather than isolated VLPO injury alone. Neuroimaging studies have linked post-stroke sleep disturbance to lesions involving the thalamus, basal ganglia, and brainstem, and to altered structural/functional connectivity that may reflect maladaptive remodeling. However, sleep–wake manifestations may vary by lesion location and stage; notably, brainstem lesions in the acute phase often present with hypersomnolence or impaired consciousness rather than prototypical insomnia (10–12).

Beyond broad sleep–wake circuitry disruption, lesion topography may shape specific downstream sleep phenotypes via comorbid sleep disorders. In acute lacunar stroke, for example, smoking and lacunes in the internal capsule or pons were associated with a higher burden of sleep-related breathing disorders, which can increase nocturnal arousals and sleep fragmentation. Because sleep-related breathing disorders primarily disrupt sleep through recurrent respiratory events, associated micro-arousals, and sleep fragmentation, insomnia complaints in this context may often reflect secondary consequences of fragmented, non-restorative sleep rather than insomnia per se; targeted screening (when feasible) is particularly relevant in topography- and risk-factor–enriched subgroups, both to avoid misattribution of symptoms and to enable mechanism-aligned management (13).

Heterogeneity implication: patients with prominent sleep fragmentation or difficulty maintaining sleep may have stronger contributions from network disruption and altered arousal stability, although lesion-symptom relationships remain probabilistic rather than deterministic.

### 2.2 Neurotransmitter and circadian dysregulation

Stroke may perturb neurotransmitter systems relevant to sleep initiation and maintenance, including serotonin (5-HT), gamma-aminobutyric acid (GABA), glutamate, and melatonin. Alterations in rhythmic secretion and homeostatic balance can shift sleep propensity and reduce inhibitory “sleep-promoting” tone. Associations have been reported between lower 5-HT and increased sleep initiation/maintenance difficulty, reduced nocturnal melatonin and poorer sleep quality early after stroke, and lower GABA levels correlating with higher insomnia severity. Circadian disruption may also be amplified during hospitalization and rehabilitation by prolonged bed rest, reduced daytime mobility, insufficient light exposure, and irregular ward routines. Although these factors are unlikely to represent the sole cause of PSI in most patients, they may meaningfully exacerbate circadian misalignment and insomnia symptoms (14–16).

Heterogeneity implication: some patients present with delayed sleep phase, irregular sleep–wake patterns, or prominent sleep-onset complaints that may reflect circadian misalignment and weakened homeostatic drive.

### 2.3 Neuroinflammation and immune dysregulation

Post-stroke inflammation—through microglial activation and peripheral immune responses—may disrupt sleep–wake homeostasis. Inflammatory mediators such as interleukin 6(IL-6), tumor necrosis factor alpha (TNF-*α*), and C-reactive protein (CRP) can influence central sleep regulatory circuits and interact with circadian signaling. Observational work has linked inflammatory marker levels during rehabilitation with nocturnal awakenings, and IL-6 has been proposed as a potential predictor of PSI. Inflammation may also promote sympathetic activation and alter hypothalamic sleep-promoting neuronal activity, contributing to fragmented sleep (17–19).

Heterogeneity implication: patients with systemic inflammatory burden or comorbid metabolic/vascular dysregulation may exhibit persistent sleep disruption and non-restorative sleep despite apparently adequate time in bed.

### 2.4 Activation of the stress system

Stroke represents a major physiological and psychological stressor. This section integrates endocrine and neural aspects of stress-related hyperactivation in post-stroke insomnia, including HPA-axis dysregulation and autonomic imbalance. Persistent activation of the HPA axis with elevated or dysregulated cortisol secretion can reduce slow-wave sleep and increase nocturnal awakenings, fostering a hyperarousal state. Autonomic imbalance and sustained sympathetic activation after stroke have been associated with lighter sleep and greater fragmentation (20–23).

Heterogeneity implication: patients with pronounced arousal symptoms (racing thoughts, nocturnal vigilance), frequent awakenings, and anxiety-related sleep maintenance problems may reflect dominant contributions from HPA–autonomic hyperactivation, often intertwined with mood symptoms.

### 2.5 Hemodynamic disturbance and neurovascular unit dysfunction

Beyond discrete lesions, post-stroke cerebral autoregulation and neurovascular unit integrity may be impaired. Sleep is accompanied by physiological changes in perfusion and autonomic tone; disrupted nocturnal hemodynamic regulation could affect perfusion in sleep-relevant regions and destabilize sleep continuity. Some studies report attenuated hemodynamic regulation during sleep in stroke populations, including altered blood flow patterns and increased awakenings during rapid eye movement (REM) sleep. Endothelial dysfunction disrupted nitric oxide signaling, and neurovascular injury may further impair sleep-related perfusion dynamics (24–26).

Heterogeneity implication: this domain may contribute to fluctuating sleep continuity and vulnerability to nocturnal awakenings, especially in patients with unstable blood pressure, vascular stiffness, or autonomic dysregulation.

### 2.6 Psychosocial and comorbid drivers

Functional limitation, communication impairment, rehabilitation demands, and environmental disruption can precipitate anxiety and depression, which show bidirectional links with insomnia and can maintain symptoms over time. Concurrent pain, nocturia/urgency, and sleep-disordered breathing may directly increase arousals and sleep fragmentation (27–30). When sleep-disordered breathing is present, symptoms may include loud snoring, witnessed apneas, nocturnal gasping, non-restorative sleep, and daytime sleepiness or fatigue (31). In stroke survivors, these manifestations may overlap with insomnia complaints, particularly sleep-maintenance difficulty and unrefreshing sleep, highlighting the importance of targeted screening.

Heterogeneity implication: in many patients, PSI is maintained by a “dominant driver” (e.g., pain, nocturia, obstructive sleep apnea (OSA), mood symptoms). Failure to identify and treat these contributors can lead to apparent treatment resistance.

### 2.7 Integrative model: hyperarousal as a convergent feature

Across domains, PSI can be conceptualized as a state of sleep–wake imbalance in which structural/network vulnerability, circadian and neurotransmitter disruption, inflammatory signaling, and HPA–autonomic activation converges on hyperarousal and reduced sleep stability. Psychosocial stressors and comorbid symptoms further reinforce this cycle. Even when the clinical complaint is similar, the dominant mechanism mix may differ substantially across individuals, providing a rationale for mechanism-informed phenotyping and stratified care.

## 3 Mechanism-informed management and objective quantification

Current PSI management often mirrors general insomnia strategies, but stroke-specific limitations (cognitive deficits, fall risk, polypharmacy, comorbidities) and the need for coordinated rehabilitation care create unique challenges.

### 3.1 Current treatments and practical constraints

Behavioral therapy, especially cognitive behavioral therapy for insomnia (CBT-I), should be regarded as the preferred first-line treatment for chronic insomnia and, whenever feasible, the preferred initial approach for persistent post-stroke insomnia. In stroke survivors, feasibility may be limited by aphasia, executive dysfunction, fatigue, and reduced access to trained therapists, potentially lowering adherence and effectiveness in routine rehabilitation settings (32, 33).

Pharmacological treatment should generally be considered a second-line or adjunctive option, particularly when CBT-I is not feasible, is insufficient, or is needed as a short-term bridge. Benzodiazepines and non-benzodiazepine hypnotics may improve sleep onset or maintenance in the short term, but concerns include tolerance, dependence, cognitive effects, daytime somnolence, and increased fall risk—issues particularly salient in stroke survivors (8, 34). Melatonin may be particularly relevant in patients with circadian misalignment, reduced nocturnal melatonin secretion, or prominent sleep-onset difficulty; however, the current evidence in post-stroke insomnia remains limited and heterogeneous (15). Dual orexin receptor antagonists may be considered as one potential option among others in selected patients, particularly when hyperarousal-related sleep difficulty is prominent, but stroke-specific evidence remains limited and available follow-up is relatively short (35). Overall, most hypnotic agents, including newer compounds, lack robust long-term safety data in post-stroke populations; therefore, when used, they should be individualized, prescribed at the lowest effective dose, used for the shortest feasible duration, and regularly re-evaluated.

Neuromodulation and rehabilitation-related interventions (including transcranial magnetic stimulation, vagus nerve stimulation, and acupuncture) have gained attention (36–38). Acupuncture trials for PSI have increased and often report improvements in subjective outcomes, such as the Pittsburgh Sleep Quality Index (PSQI) (39). However, interpretation is constrained by protocol heterogeneity, challenges in control/blinding, inconsistent outcomes, and limited follow-up, which reduce reproducibility and the strength of clinical recommendations.

### 3.2 Why objective sleep metrics matter

Most PSI studies rely heavily on subjective scales (PSQI, Insomnia Severity Index (ISI)). While clinically meaningful, questionnaires alone cannot fully capture multidimensional sleep changes (sleep efficiency, awakenings, sleep onset latency, sleep continuity, and stage distribution). Without objective measures, it remains difficult to: Verify whether an intervention changes sleep physiology versus symptom perception, link improvement to specific mechanistic domains (e.g., reduced arousal vs. improved circadian alignment), and identify patient subgroups most likely to benefit.

Polysomnography is the gold standard, but it is resource-intensive and often impractical during rehabilitation. Actigraphy and wearable sleep monitoring may offer scalable longitudinal assessment, enabling trend tracking and dynamic evaluation in real-world settings. A pragmatic framework combining subjective symptoms with objective metrics can support more actionable phenotyping and more interpretable evidence.

### 3.3 A pragmatic stratified pathway

A mechanism-informed approach can be operationalized without requiring advanced biomarkers in every patient. In practice, dominant drivers should be identified through structured clinical assessment, including detailed sleep history, sleep diaries, standardized questionnaires, and objective monitoring when appropriate. Actigraphy or wearable monitoring may help characterize circadian or behavioral dysregulation, whereas polysomnography is particularly useful when sleep-disordered breathing or other comorbid sleep disorders are suspected; targeted laboratory or imaging studies may be informative in selected contexts. Clinically, stratification may begin with identifying dominant drivers and matching interventions accordingly:

- Mood/stress-dominant profiles: prioritize brief psychological strategies, adapted CBT-I elements, stress reduction, and rehabilitation environment optimization, particularly when anxiety, rumination, or mood symptoms are prominent.
- Hyperarousal/sleep maintenance profiles: consider interventions targeting arousal reduction and sleep continuity (carefully selected pharmacotherapy when appropriate; structured behavioral components; neuromodulation strategies in research settings), particularly when nocturnal vigilance, frequent awakenings, or difficulty returning to sleep predominate.
- Comorbidity-dominant profiles: systematically screen for and manage pain, nocturia, sleep-disordered breathing, and medication-related sleep disruption; treating these contributors may yield outsized improvements, especially when clinical history suggests snoring, witnessed apneas, nocturnal discomfort, or medication-related sleep disturbance.
- Circadian/behavioral dysregulation profiles: emphasize daytime activity scheduling, light exposure, and consistent sleep timing, supported by wearable-derived feedback when available, particularly when irregular sleep timing, low daytime activity, or reduced light exposure are evident.

Standardized protocols paired with objective outcomes can strengthen mechanistic interpretability for neuromodulation-oriented approaches (including acupuncture) and facilitate evidence translation.

## 4 Discussion

### 4.1 Controversies

Key controversies include whether PSI represents a distinct stroke-related syndrome versus a symptom cluster primarily driven by comorbidities, and whether improvements on subjective scales reliably reflect restoration of sleep physiology. Additional debate surrounds the evidentiary strength and generalizability of neuromodulation and acupuncture studies, given variability in protocols and methodological constraints.

### 4.2 Research gaps

Current PSI research is limited by (i) inconsistent definitions, assessment windows, and thresholds; (ii) predominance of cross-sectional or short-term studies with limited causal inference; (iii) insufficient control of confounders (mood symptoms, stroke severity, comorbidity burden, medication effects); (iv) underuse of objective sleep metrics; and (v) intervention heterogeneity with challenges in control design, blinding, and adverse event reporting.

Consistent with these field-level limitations, the present narrative review also has several constraints. This review is narrative in nature and was not designed as a systematic review or meta-analysis; therefore, study selection may be subject to selection bias and incomplete coverage. Objective sleep assessments and longer follow-up are underrepresented in the current evidence base, constraining mechanistic inference and conclusions about durability. Finally, much of the available evidence is observational, and residual confounding by mood symptoms, stroke severity, comorbidities (e.g., sleep-disordered breathing), and medication effects remains a key limitation when interpreting associations and treatment effects.

### 4.3 Potential developments

Near-term priorities include establishing standardized PSI definitions and core outcome sets that combine subjective and objective sleep measures; deploying scalable objective phenotyping (actigraphy/wearables) for longitudinal monitoring; integrating selected mechanistic endpoints (inflammatory, autonomic, imaging) in rigorous designs; and developing stratified management pathways that explicitly target dominant drivers and improve reproducibility across studies.

Against this backdrop, an actionable near-term direction is comparative phenotyping of PSI across ischemic stroke subtypes, particularly lacunar versus non-lacunar stroke, given differences in vascular pathology, clinical features, and prognosis. Lacunar infarcts often show a distinct pathophysiology and clinical profile and may have a relatively favorable short-term prognosis compared with other acute cerebrovascular diseases, underscoring the value of subtype-stratified analyses (40). Intracerebral hemorrhage, however, may require separate interpretation in future PSI studies, particularly in the acute phase, when impaired consciousness or hypersomnolence may be more prominent than insomnia.

Besides, cognitive impairment is a core feature of cerebrovascular disease and may be both a contributor to and a consequence of disturbed sleep after stroke. Small-vessel disease burden—often captured by WMH volume and lacunes—has been associated with worse post-stroke cognitive functioning across domains (41); moreover, microstructural white-matter integrity has been linked to processing speed performance, supporting a plausible pathway by which SVD-related network injury intersects with sleep disruption and daytime function (42). In this context, PSI phenotyping that includes cognitive screening (particularly executive control/processing speed) may help identify subgroups in whom treating dominant drivers of nocturnal fragmentation (e.g., SRBD, nocturia, pain, hyperarousal) could yield downstream benefits for rehabilitation engagement and cognition.

Such designs, paired with harmonized subjective–objective outcomes and appropriate adjustment for vascular risk factors, could clarify subtype-specific PSI trajectories and support stratified, mechanism-aligned interventions. Future studies should further clarify the long-term trajectories of distinct PSI phenotypes, as persistent fragmentation-, circadian-, hyperarousal-, or comorbidity-dominant patterns may have different implications for recovery and prognosis. Longitudinal studies incorporating standardized phenotypic definitions and objective follow-up measures are needed to better define these trajectories and their therapeutic relevance (43).

## 5 Conclusion

PSI is common after stroke and clinically relevant for rehabilitation participation, quality of life, and possibly long-term outcomes. Evidence supports a multidimensional etiology involving lesion/network disruption, neurotransmitter and circadian dysregulation, neuroinflammation, HPA axis and autonomic hyperarousal, neurovascular dysfunction, and psychosocial/comorbid contributors. Current management often extrapolates general insomnia strategies and is constrained by comorbidity burden, intervention heterogeneity, and limited objective quantification. Advancing PSI care will likely require standardized definitions, combined subjective–objective phenotyping, and mechanism-informed stratified interventions that prioritize dominant drivers and support durable, interpretable outcomes.

Future work should prioritize harmonized PSI definitions and core outcome sets that combine symptom scales with objective sleep metrics, alongside adequately powered longitudinal cohorts. Stratified, mechanism-informed trials that target dominant drivers (e.g., sleep-related breathing disorders, pain, nocturia, hyperarousal, circadian misalignment) and incorporate stroke subtype/topography will be critical to improve reproducibility and translation into stroke rehabilitation practice.
